# Kinetic and Dynamic Computational Model-Based Characterization of New Proteins in Mice: Application to Interferon Alpha Linked to Apolipoprotein A-I

**DOI:** 10.1371/journal.pone.0042100

**Published:** 2012-07-27

**Authors:** Zinnia Patricia Parra-Guillen, Jessica Fioravanti, Jose Medina-Echeverz, Celia Gomar, Nuria Ardaiz, Iñaki F. Troconiz, Pedro Berraondo

**Affiliations:** 1 Department of Pharmacy and Pharmaceutical Technology, School of Pharmacy, University of Navarra, Pamplona, Navarra, Spain; 2 Division of Hepatology and Gene Therapy, Centre for Applied Medical Research (CIMA), Pamplona, Navarra, Spain; University of Milan, Italy

## Abstract

Interferon alpha linked to apolipoprotein A-I has been recently proposed as an improved interferon-based therapy. In the present study, we aimed to develop a computational model to gain further insight into the *in vivo* behaviour of this new fusion protein. In order to facilitate *in vivo* evaluation of interferon and the fusion protein without altering their biological properties, green fluorescent protein was incorporated into their structures. Kinetic and dynamic behaviour of both compounds was successfully described after plasmid hydrodynamic administration and *in situ* synthesis of the studied proteins. Results from the modelling exercise showed that apolipoprotein A-I conferred a modified kinetic behaviour, varying molecule distribution and prolonging half-life without altering liver dynamic performance. However, differences in the gene expression activity were observed at brain level between both compounds. Those differences could be explained by modifications in the dynamic, but also in the biodistribution properties, which would be worth evaluating in future experiments. Therefore, the modelling approach provided a global comprehension of a complex system and allowed us to compare the *in vivo* behaviour of both compounds and to identify critical aspects that might be important to understand the system better and suggests a need for new model-based experiments.

## Introduction

Interferons (IFN) are a family of cytokines widely used in clinics owing to their antiproliferative, antiviral and immunomodulatory properties [1], [2]. IFNα was first proved to be beneficial in the treatment of hepatitis C in 1986 [3], and, although the rate of success in monotherapy was low (12–16%), the addition of the antiviral agent ribavirin significantly enhanced the therapeutic response (35–40%) [4]. Further improvements were achieved when pegylated interferons were put on the market. These new molecules showed a better kinetic profile and an increased rate of therapeutic response, and thus became, in combination with ribavirin, the standardized regime used in clinical medicine for chronic hepatitis C [5], [6]. Nevertheless, the rate of sustained viral response in chronic patients is still insufficient (54–56%) [7], [8] and the severity of some side effects, such as neutropenia, thrombocytopenia [9], [10] and specially psychiatric disorders like depression, greatly limit their use in clinical practice [10], [11], being necessary to discover new therapeutic agents.

Different strategies have been proposed to improve interferon-based therapies (reviewed in [12]). One molecule recently developed is a potent immunostimulatory fusion protein, termed IA, obtained when IFNα is covalently attached to apolipoprotein A-I (ApoAI) [13], major component of high-density lipoproteins (HDLs) [14]. The presence of ApoAI in this new molecule has proven not only to facilitate the incorporation of both entities into the circulating HDLs, which translates into increased stability and prolonged half-life of IA, but also to provide a different *in vivo* biodistribution profile**,** with promising liver-targeting qualities [15]–[17].

HDL uptake in the brain is a highly regulated process [18], and facilitated transfer through the blood-brain barrier (BBB) has been previously described for molecules bound to ApoAI [19]. The different brain distribution between IFNα, which is thought to enter into the brain through passive diffusion [20], [21], and IA, could be expected to translate into limited IA brain entry, and therefore central nervous system-related side effects, at high doses like those used in clinical practice.

Kinetic/dynamic modelling has proven to be an interesting approach to describe and understand the *in vivo* behaviour of therapeutic molecules, providing a useful tool to explore different mechanisms of action, new scenarios and to optimize experimental designs *in silico* [22]–[24]. A few examples where a modelling approach has been followed to evaluate the kinetic of different non-viral or viral vectors in order to improve their *in vivo* behaviour [25], [26], or to study the dynamic (efficiency) of vectors [27], [28], can be found in the literature. However and despite its advantages, its use in the gene therapy field is still limited [29], especially due to the amount of experimental data and computational resources needed, and our knowledge non-integrated kinetic/dynamic model has been develop so far in preclinical settings.

The final aim of the study is to evaluate the kinetic and dynamic properties conferred by the incorporation of ApoAI to therapeutic molecules such as IFNα through mathematical modelling. Nevertheless, model-based is a quantitative approach, and techniques to quantify IFNα might not be sensitive enough or might provide an unacceptable background due to detection of endogenous protein. Therefore, in order to facilitate the quantification of this protein *in vivo*, green fluorescent protein (GFP) sequence was added to the plasmid codifying for the IA protein. GFP is a fluorescent protein widely used in molecular biology. Among the known properties, we can highlight its great versatility and its ability to preserve the native function of proteins after their fusion to both the C and N-terminal region of GFP [30]. Accordingly, the IA codifying plasmid with the GFP sequence in between (IFNGFPApo), and the interferon-GFP plasmid (IFNGFP) or the apolipoprotein AI fused to GFP (GFPApo) as comparison molecules, were cloned in order to evaluate the differential properties conferred by ApoAI.

Here, we demonstrate how a computational semi-mechanistic model can be used to quantitatively characterize the kinetic and dynamic properties of IFNGFPApo and IFNGFP, showing a modified kinetic profile for both molecules without modifying IFNα efficacy in liver. However, dynamic differences between these two compounds were found in brain, suggesting the need for new experiments to clarify the behaviour of the system at this level.

## Results

### Evaluation of pGFPApo, pIFNGFP and pIFNGFPApo

GFP molecule was selected for this project due to its known capacity to bind molecules to both GFP terminal regions of the protein without modifying the structure of any of the compounds implied, and therefore conserving their native properties. Nevertheless, GFP fluorescence, antiviral IFNα activity and the capacity of ApoAI to be transported inside the HDLs were evaluated to confirm whether the biological properties of each of the three proteins were retained.

**Figure 1:**
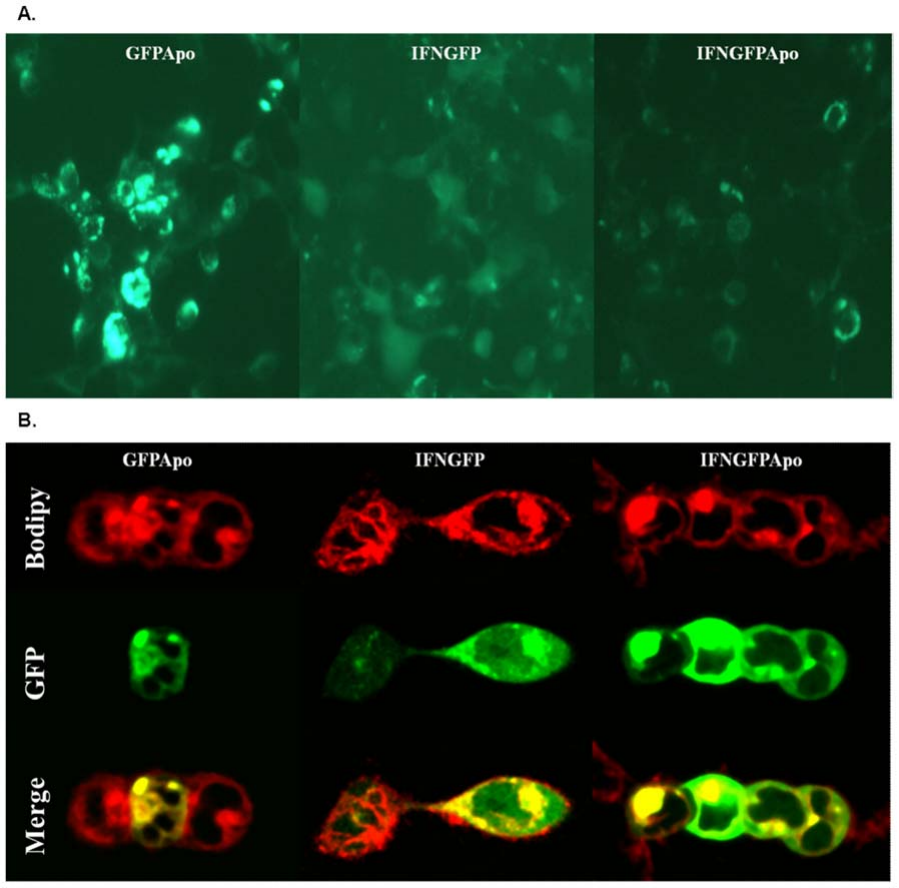
Evaluation of GFP activity by microscopy analysis. 2×10^5^cells per well were seeded and transfected with pGFPApo, pIFNGFP and IFNGFPApo complexed in PEI 25000(kDa). 24 hours after transfection of GFPApo (left), IFNGFP (center) and IFNGFPApo (right) **A.** fluorescence was evaluated by fluorescence microscopy or **B.** cells were dyed with BODIPY TR and analyzed by confocal microscopy.

To assess the bioavailability of GFP, 293T cells were transfected with pGFPApo, pIFNGFPApo or pIFNGFP, and observed through fluorescence microscopy after 24 h of incubation (**Fig. 1A**). Green fluorescence was detected in all scenarios, although different distribution patterns could be observed. While IFNGFP was homogeneously distributed inside the cell, GFP bound to IA presented a heterogeneous distribution with apparent fluorescence accumulation around the cytoplasmatic cell borders, like those observed in GFPApo. To gain further insight into the differential distribution, transfected cells were stained with a red-fluorescent dye to visualize the Golgi apparatus and analyzed by confocal microscopy (**Fig. 1B**). IFNGFPApo also presented an intermediate distribution pattern between GFPApo, only detected in the Golgi and absent from the nucleus, and IFNGFP, homogenously located in both nucleus and Golgi.

**Figure 2:**
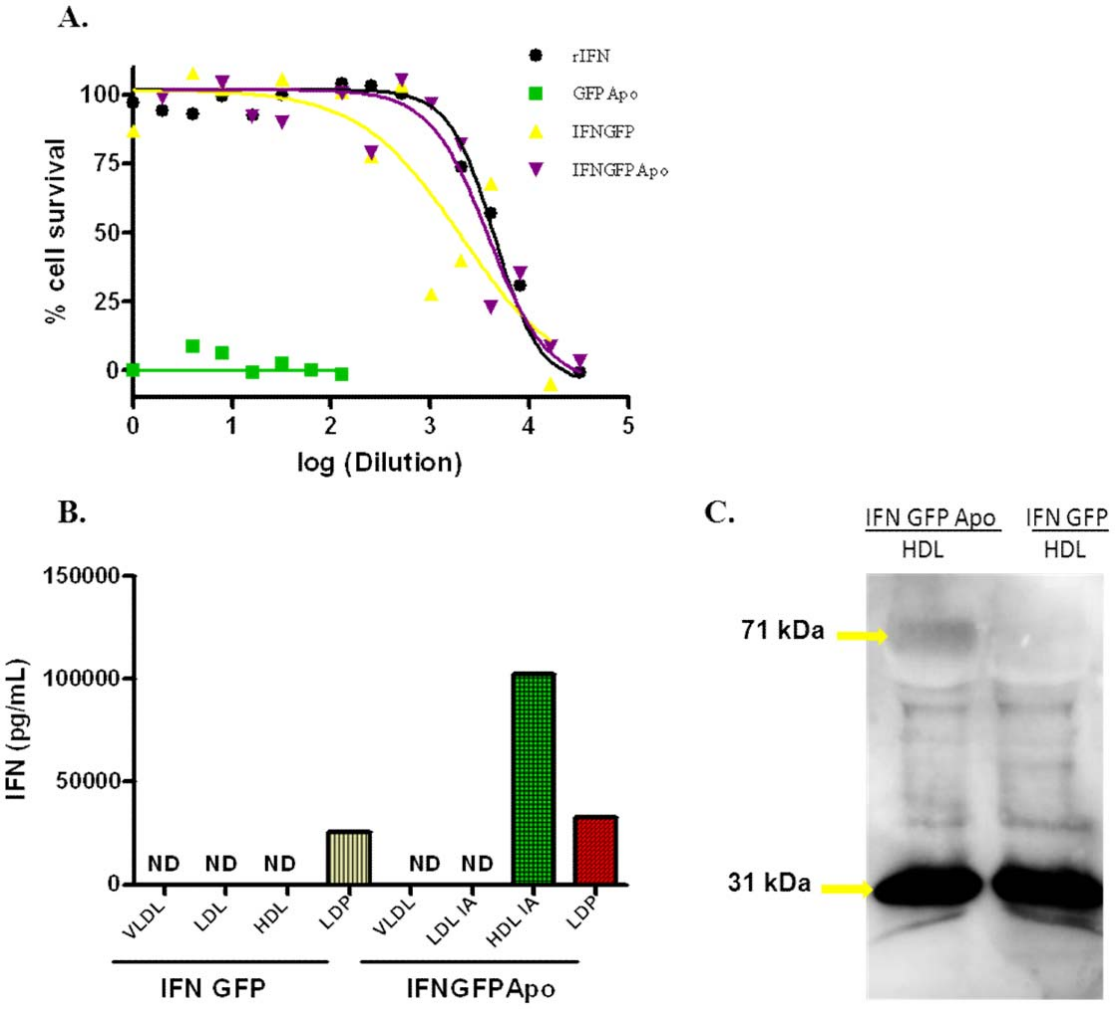
Evaluation of IFNα and ApoAI biological properties. **A.** recombinant IFN (rIFN), GFPApo, IFNGFP and IFNGFPApo antiviral efficacy against encephalomyocarditis virus expressed as percentage of surviving cells against molecule dilution. **B.** Determination by ELISA of the IFNα content of the different plasma lipoprotein fractions obtained after 24 h of hydrodynamic injection of 20 µg IFNGFP or IFNGFP plasmid. Pooled samples of three mice per group were used. **C.** Western blot against ApoAI of HDL plasma fractions obtained as previously described.

Supernatants obtained after 293T cell transfection were used to evaluate the capacity of the three synthesized proteins to protect L929 cells against encephalomyocarditis virus infection. As expected, only those molecules where IFNα was present in the structure were able to protect L929 cells against the viral infection and subsequent cell death (**Fig. 2A**). Moreover, both, IFNGFP and IFNGFPApo presented a similar value of drug concentration needed to inhibit 50% of cellular death (IC_50_) (p>0.05) (Log IC_50_ equal to 3.32 and 3.59 respectively).

Regarding the preservation of the native ApoAI function, 20 µg of either pIFNGFP or pIFNGFPApo were administered to the BALB/c mice strain (n = 3 mice per group) through hydrodynamic injection. 24 hours later, blood samples were obtained, the different plasma lipoprotein fractions were separated until the last fraction was considered to be free of lipoproteins (LDP, lipoprotein-depleted plasma) and IFNα content was measured in each of the fractions. In the case of pIFNGFP, IFNα presence was only detected in the LDP fraction (**Fig. 2B**), and not in the very low density lipoprotein (VLDL), low density lipoprotein (LDL), or high density lipoprotein (HDL) fractions. Hence, we concluded that IFNGFP was not able to bind to the plasma lipoproteins. Only when ApoAI was present in the structure was IFNα also detected in the HDLs fraction (**Fig. 2B**). To ensure that solely IFNGFPApo was able to travel along with HDLs, western blotting against ApoAI over the HDL fraction of both molecules was performed. A band around 31 kDa corresponding to endogenous ApoAI could be observed in both fractions, yet only the IFNGFPApo fraction presented a higher band (around 71 kDa) representing the triple fusion protein bound to HDL (**Fig. 2C**).

In conclusion, three plasmids codifying for three novel fusion proteins were obtained without altering the three-dimensional structure of any of the molecule components, and consequently, retaining the antiviral activity of IFNα (therapeutic drug), the capacity of ApoAI molecule to travel along with the HDLs and the GFP structure introduced to increase the sensibility and specificity of the quantification method. Therefore, IFNGFP and IFNGFPApo plasmids could be used to evaluate *in vivo* the kinetic and dynamic differences between the main molecules of interest: IFNα and IA.

### Kinetic Model

*In vivo* performance of IFNGFP and IFNGFPApo was evaluated, focusing especially on hepatic production, serum profiles, and brain distribution. The model was developed following a step-wise approach, where mRNA levels and liver (l), serum (s) and brain (br) protein measurements were added sequentially to the model.

**Figure 3:**
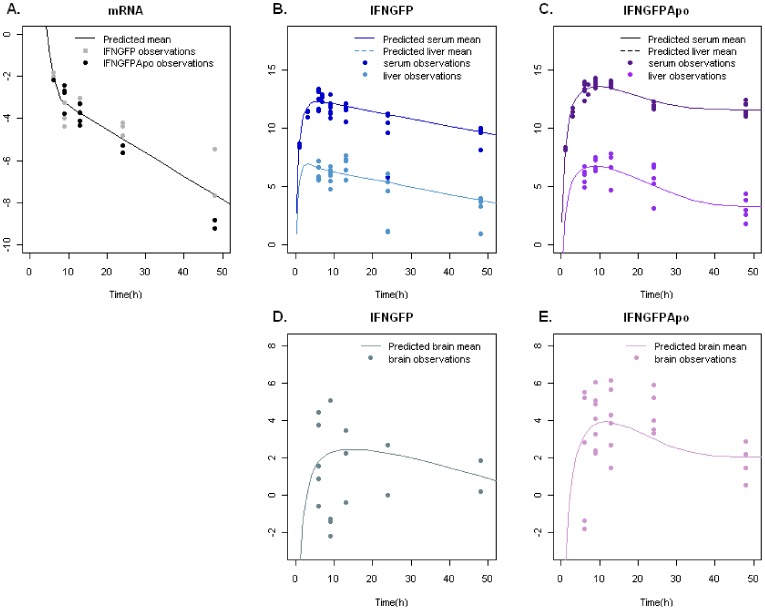
Mean concentration-time profiles of mRNA and proteins in liver, serum and brain. 20 µg of either IFNGFP or IFNGFPApo plasmids were administered by hydrodynamic injection. At the selected time points, a group of mice was sacrificed and the liver, the brain and a serum sample were obtained to quantify levels of GFP protein or mRNA. Observed (points) and predicted levels (lines) were plotted versus time for (**A**) mRNA gene expression of both molecules in liver, (**B**) serum and liver GFP levels after IFNGFP or (**C**) or IFNGFPApo plasmid administration, and (**D**) brain levels reached with IFNGFP or (**E**) IFNGFPApo. Brain and liver levels are expressed in GFP pg/mg protein; serum levels are expressed on GFP pg/mL units and mRNA on gene expression units in log scale.

**Figure 4:**
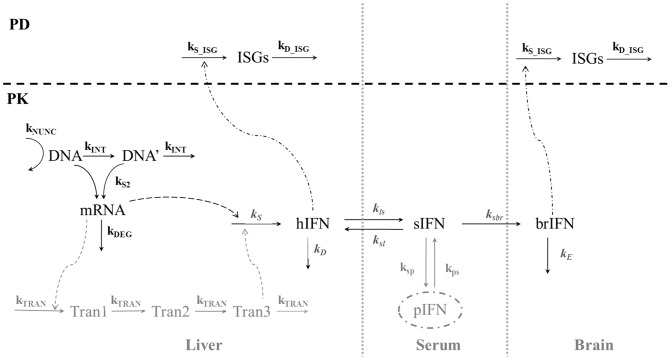
Schematic representation of the PK/PD model. IFNGFP model is represented in black. IFNGFPApo modifications over the basic IFNGFP model are drawn in grey. Common model process equivalences: k_NUNC_, first order rate constant of degradation of DNA; k_INT_, first order rate constant controlling the formation and elimination of DNA’; k_S1_ and k_S2_, first order rate constants of expression of mRNA from DNA and DNA’, respectively; k_DEG_, first order rate of degradation of mRNA; k_TRAN_, first order constant rate of transit; k_S_ and k_D_, first order rate constants of synthesis and degradation of the hepatic protein; k_ls_ and k_sl_ first order rate constants of distribution between liver and serum compartments; k_sbr_, first order rate constants of brain input; k_E_, first order rate of degradation of brain IFN; k_S_ISG_ and k_D_ISG_, the first order process of synthesis and degradation in liver or brain. Specific parameter of the IFNGFPApo model: k_sp_ and k_ps_, distribution rate constants between central serum compartment and peripheral compartment, respectively.

The five panels in Figure 3 show that the final model represented schematically in Figure 4 and mathematically by the set of equations 1–11 captured well the concentration versus (vs) time profiles in liver, serum, and brain of mRNA, IFNGFP, and IFNGFPApo. Kinetic parameters are summarized in Table 1. Precision of model parameters, computed as the ratio between the standard errors (listed in Table 1 in parenthesis) and the estimate of the parameters were in general below 50% indicating that model parameters were indeed identifiable given the model and the available data.

Interestingly, the kinetics of hepatic transcription from pDNA to mRNA for the two molecules could be described with the same model and same set of model parameters (Table 1).

**Table 1:** Kinetic parameters.

Parameters	IFNGFP^a^	IFNGFPApo^a^
k_NUNC_ (h^−1^)	1.60 (0.0049)	
k_INT_ (h^−1^)	0.112 (13.21)	
k_S1_ (gene expression* h^−1^*gene expression^−1^)	1080 (15.93)	
k_DEG_ (h^−1^)	2.12 (1.24)	
k_S2_ (gene expression* h^−1^*gene expression^−1^)	2.96 (16.32)	
k_S_ (pg* h^−1^*gene expression^−1^)	769 (0.4)	53600 (1.57)
k_D_ (h^−1^)	22.4 (2.35)	0.0773 (8.02)
k_TRAN_ (h^−1^)	1.45 (0.86)	0.266 (2.75)
k_ls_ (h^−1^)	120 (11.75)	1720 (17.03)
k_sl_ (h^−1^)	0.406 (11.85)	0.420 (9.81)
k_sp_ (h^−1^)	NA	1.54 (6.95)
k_ps_ (h^−1^)	NA	0.00994 (0.5)
k_sb_ (h^−1^)	1.08×10^−6^ (40.00)	3.75×10^−5^ (19.47)
k_b0_ (h^−1^)	0.118 (23.22)	0.509 (39.10)

NA: non applicable.

a Parameter estimates are listed together with the corresponding coefficient of variation [CV(%)] calculated as the ratio between the standard error and the estimate of the parameter.

On the contrary, a poor model performance was observed when a common model structure and parameter estimates were attempted to describe liver and serum kinetics of both proteins simultaneously. Therefore, each molecule was independently analysed. IFNGFP profiles were successfully described using a chain of three transit (signalling) compartments to delay liver IFNGFP synthesis (dMVOF = 18 when incorporating one extra parameter and compared with no delay, p<0.001) and reversible mass transfer between liver and serum compartments (equations 6–7) (**Fig. 3B**). The same model structure, though with a different set of parameters, was applied to the IFNGFPApo data from liver and serum also observing an improvement in the fit when a delay in the protein synthesis was introduced (dMVOF = 73 when incorporating one extra parameter, p<0.001) (**Fig. S1A**). However, the mean transit time estimated for IFNGFPApo was significantly different from the IFNGFP estimated one (15 h versus 2.76 h respectively) (p<0.001) suggesting a slower synthesis process. A further improvement in the IFNGFPApo model performance was observed when including an extra compartment for protein distribution outside the serum (central) compartment (dMVOF = 19 when incorporating two extra parameters p<0.01) (**Fig. S1B**). During model building, degradation of both proteins from the serum compartment was also assayed but it did not improve model description (p>0.05).

The concentration of both proteins in the liver was similar, as can be seen in panels 3b and 3c, but in serum, the difference in levels was more apparent showing an increase in serum half-life (Figure S2 allows a better comparison of the rates of elimination from serum). Serum half-life is a parameter related to both, elimination and disposition processes. The area under the curve was greater for the case of sIFNGFPApo, suggesting a lower clearance. This fact, together with the existing differences in the distribution properties of both proteins, supported by the need of a peripheral compartment in the IFNGFPApo case, would be responsible for an increase in the terminal half-life in serum. However, as that total amount of synthesized protein remains unknown, it was not possible to calculate an apparent volume of distribution.

Results from the brain analysis indicated a faster turnover brain kinetics regarding IFNGFPApo. The slower first order rate constant of exit from the brain compartment was four-fold lower for brIFNGFP, and although it reached not statistical significance, it pointed towards an extended distribution, as will be discussed later in conjunction with the dynamic analysis.

### Dynamic Model

Interferon-stimulated genes (ISGs) are genes selectively activated by interferon, which can therefore be used as a measure of interferon activity. Two main organs were selected regarding their clinical interest: liver, main target organ of the therapy, and brain, where important and greatly limiting side effects arise with the current standard of care. ISG15 and 2′-5′ OAS genes were selected as indirect measurements of IFNα activity.

**Figure 5:**
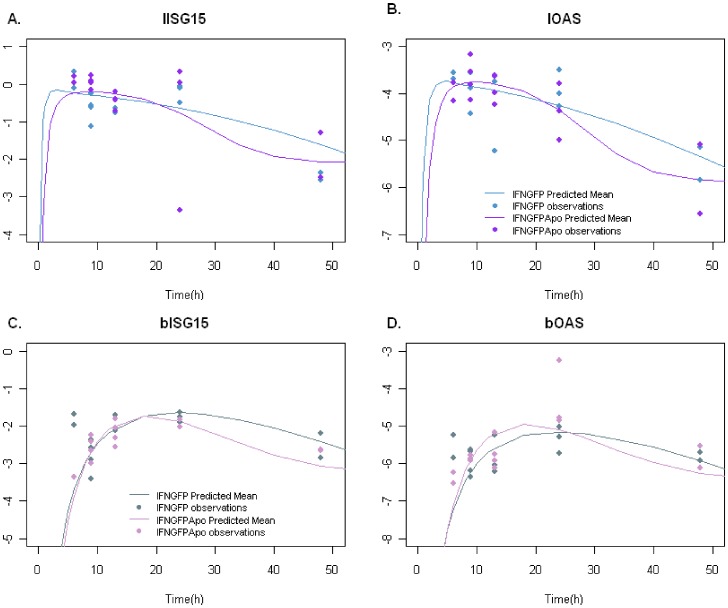
Mean concentration-time profiles of Interferon Stimulated Genes (ISGs) in brain and liver. Mean concentration-time profiles of Interferon Stimulated Genes (ISGs) in brain and liver. Observed (points) and predicted (lines) levels were plotted versus time for (**A–B**) mRNA expression of OAS or ISG15 interferon stimulated genes respectively in liver or (**C–D**) in brain. ISGs are expressed in gene expression units in log scale.

In the liver and for the two genes studied, the synthesis was best described with a saturation (non-linear) model of the form k_S_x[IFN/(IFN+IFN_50_)] (AIC value equal to 217.8 and 210.7 for OAS and ISG15 respectively) instead of the linear model k_S_xIFN ((AIC value equal to 230.0 and 237.0 for OAS and ISG15 respectively). For each gene, proteins profiles were successfully described considering the same gene expression efficiency for both compounds (**Figs. 5A–B**). Model performance did not significantly improve if different sets of model parameters were estimated (dMVOF equal to 2 and -9 points for OAS and ISG15 respectively when incorporating two new parameters, p>0.05). Model parameters are summarized in Table 2.

**Table 2:** Dynamic parameters.

Genes	Parameter	LIVER		BRAIN	
		IFNGFP^a^	IFNGFPApo^a^	IFNGFP^a^	IFNGFPApo^a^
ISG15	k_S_ISG_ (gene expression*h^−1^)^b^	491 (7.31)		2.05×10^−3^(21.12)	6.08×10^−4^(17.11)
	k_D___ISG_ (h^−1^)	491 (7.31)		0.127 (24.33)	
	IFN_50_ISG15_ (pg*mL^−1^)	151 (0.47)		NA	NA
OAS	k_S_OAS_ (gene expression*h^−1^) b	0.0366 (81.69)		7.37×10^−5^ (23.47)	2.45×10^−5^ (23.47)
	k_D_OAS_(h^−1^)	1.18 (87.29)		0.101 (23.76)	
	IFN_50_OAS_ (pg*mL^−1^)	255 (43.14)		NA	NA

a Parameter estimates are listed together with the corresponding coefficient of variation [CV(%)] calculated as the ratio between the standard error and the estimate of the parameter.

b Brain parameter units in gene expression*mL*pg^−1^*h^−1^.

The same approach was followed to describe brain ISG expression levels; however, it was found that expression efficiencies differed between IFNGFP and IFNGFPApo for the two genes (Table 2) (dMVOF equal to 15.2 and 35.1 points for OAS and ISG15 respectively when considering two different synthesis rates, p<0.001). Non-linear models for gene expression in brain were also tested, but they did not improve model performance (AIC value equal to 211.6 and 210.4 for OAS and ISG15 respectively, but with standard errors greater than 100%) compared with the linear approach (AIC value equal to 219.3 an 210.7 for OAS and ISG15, respectively with proper standard errors). Results indicated that IFNGFP induced gene expression more efficiently in a significant manner, since brain levels were lower (**Fig. 3D–E**) and gene levels very similar (**Figs. 5C–D**). Once the genes were expressed in brain their degradation rate constant was the same regardless of the type of protein (IFNGFP or IFNGFPApo) responsible for the synthesis (dMOVF equal to −3.5 and −0.2 points for OAS and ISG15 respectively when including one model parameter, p>0.05).

## Discussion

Hepatitis C is considered a major health issue affecting about 3% of the worldwide population. Pegylated interferon in combination with ribavirin is the current standard therapy; however, this approach shows limited treatment efficiency (around 50% of the patients) and major side effects, such as haematological or neuropsychiatric alterations, and therefore new treatment strategies are needed [5], [31]. A novel fusion protein between IFNα and apolipoprotein A-I has recently been proposed by Fioravanti *et al* [13]. This new molecule has proven not only to prolong interferon half-life, but also to decrease the activation of interferon-stimulated genes in the brain at therapeutic doses, indirectly suggesting a modification in the IFNα biodistribution profile and consequently, in its adverse events.

To further explore the biodistribution properties of this new entity, GFP coding nucleotides were incorporated into IA plasmid, seeking an increase in the sensitivity and a decrease in the limit of quantification of the method by incorporating an exogenous compound. Green fluorescence, antiviral activity and inclusion in the HDLs were evaluated to ensure that the cloning process had not altered GFP, IFNα and ApoAI characteristics. All conjugates were found to keep their native functions, and therefore IFNGFPApo and IFNGFP, as control compound, were used to study *in vivo* the biodistribution and kinetic changes produced when ApoAI was anchored to IFNα.

Protein biosynthesis can be an expensive and long process, sometimes unworthy in the early stages of drug development, when the relevance of the new therapeutic protein has not yet been clearly demonstrated. Hydrodynamic injection has been shown to be a useful technique to evaluate these new compound properties *in vivo* by administering naked plasmid, and forcing its expression *in situ* [32], [33].

A sequential kinetic model incorporating liver, blood and brain compartments was developed for IFNGFPApo and for IFNGFP. To jointly describe the mRNA kinetics of both compounds, a model previously published [28] was implemented.

A similar simultaneous fit was also assayed for hepatic and serum GFP concentrations, but different dynamic profiles were observed for each compound under study, with a delay in the hepatic and serum peak concentrations and prolonged plasma levels of IFNGFPApo. Banerjee *et al* [34] studied the biosynthesis and secretion of ApoAI, suggesting a Golgi accumulation of the protein prior release to blood flow. These results are in agreement with the observed IFNGFPApo synthesis dynamics (**Fig. 1B**), but also with the fluorescence accumulation detected when 293T cells were transfected with pIFNGFPApo (**Fig. 1A**). From a modelling perspective, these hypotheses are supported by the lower transit rate constant estimated for IFNGFPApo than for IFNGFP and the need for a peripheral compartment, which inclusion was only statistically significant in the case of the IFNGFPApo compound. Hepatic IFNGFPApo levels are controlled by the amounts present in the last transit compartment more flattened the lower the value of the transit rate constant (**Fig. S3**) and thus explaining the significantly greater IFNGFPApo estimated expression efficiency (k_S_) (Table 1), despite the equivalent mRNA and DNA kinetics and the similar liver concentrations of both compounds.

IFNGFPApo and IFNGFP fusion proteins also showed differences in the serum level transport driven by the presence of ApoAI; while the former was only detected at the lipoprotein depleted plasma, IFNGFPApo was able to travel and distribute bound to HDLs, and therefore, was mostly detected on the HDL lipoprotein fraction. This differential serum transport translated into different distribution features, supported by the need of a peripheral serum compartment to describe satisfactorily IFNGFPApo kinetics (**Fig. S1B**).

As discussed earlier, when bound to ApoAI, IFNα is able to incorporate into HDLs, molecules responsible for the transport of cholesterol from different body tissues to the liver in a process mediated by the scavenger receptor BI (SR-BI), the main receptors of ApoAI [35], [36]. Considering this prior biological knowledge, an increased liver distribution and a prolonged half-life driven by HDLs protection was initially expected after fusion between IFNα and ApoAI. Given the nature of the molecules (proteins of high molecular weight) significant differences in mechanisms of degradation were not expected, and considering that ApoAI is the only difference between both constructions, parameter differences are more likely to be explained by biological impediments to IFNGFPApo elimination or/and greater tissue distribution due to the inclusion of the ApoAI protein in the structure. These experimental hypotheses were supported from a modelling perspective by the lower elimination constant rate (0.0773 versus 24.4 h^−1^ for IFNGFP and IFNGFPApo, respectively). However, liver vectorization could not be proven, probably due to the nature of the hydrodynamic technique used for the *in vivo* experiments, since hepatic protein levels contained information from two main processes; protein expression, and drug disposition. These results pointed towards a plausible drug protection, also supported by a previous study where inclusion of ApoAI into liposomes resulted in hepatic vectorization [15]. Information gathered under different experimental conditions, such as after intravenous administration of both proteins, different amounts of DNA injected or sequential administrations would improve the validity and predictive capacity of the model and would help to confirm these hypotheses.

Another controversial point to address was brain distribution. IFNα is believed to cross BBB at low levels (less than 0.2%) through passive diffusion [37], whereas IA could follow an active transport mediated by ApoAI presence. Although active transport could not be proven for the IFNGFPApo molecule, a three times higher brain diffusion rate was found for the higher molecular weight compound (IFNGFPApo), suggesting the existence of a facilitated transport that would be mediated by ApoAI, the only difference between both compounds. Similar results were found when ApoAI [19] or other molecules with known receptors at brain level were used to increase transport of therapeutic molecules across the BBB [18], [38].

Regarding the efficacy of the two drugs, interferons are known to induce the expression of multiple genes implied not only in antiviral activities, but also in the lipid metabolism or apoptosis. Interferon-stimulated genes ISG15 and OAS were selected to evaluate the treatment effectiveness for their higher response to interferon [37], [39]. Despite the different kinetic profiles exhibited by IFNGFPApo and IFNGFP at the three analysed levels, a common dynamic model for each ISG could successfully describe the gene expression induced by IFNα in liver after either plasmid administration, suggesting that modifications introduced by ApoAI fusion did not alter its biological activity at this level. Contrary to liver, brain ISGs dynamics could not be fitted with a common model, and different synthesis rates for each gene and molecule were estimated (Table 2), IFNGFP being more efficient than IFNGFPApo. Interestingly, brain IFNGFP exit was also lower, suggesting an increased brain distribution that could also explain the apparent different gene expression efficiencies at this level. New experiments where IFNGFP and IFNGFPApo are quantified in different brain areas would be desirable to understand the system better.

In conclusion, incorporation of exogenous compounds, such as GFP, has facilitated the determination of the time-profiles of IFN and IA in three main organs: blood, liver and brain, and the development of an integrated kinetic/dynamic model. Different kinetic properties were concluded for the two compounds, estimating a prolonged half-life and distribution properties. Regardless kinetic differences, each of the gene profiles was successfully described by the same set of parameters for both molecules, indicating a preservation of the native IFNα activity in the target organ, although not in the brain, where more information would be needed before drawing any conclusion.

Our model aims to mathematically describe the different biological processes that take place in the body after plasmid administration (mRNA transcription, protein synthesis, distribution and so on) in a simplified way. Hence it is expected that this same model structure can help to describe the behaviour of other drugs, though with different sets of parameters, that account for their specific pharmacokinetic and pharmacodynamics characteristics. As an example, the transcription model applied to our data was used previously to successfully describe the luciferase activity [28].

Multiple time data analysis provides a greater amount of information, but also complicates global data interpretation. The modelling approach incorporated in the current study allows us to design experiments and to integrate all available information and hence permits us to estimate parameters with a certain biological relevance. Clearly, the integration of information has allowed us to scrutinize kinetic and dynamic differences between the two compounds and to support them from a biological point of view, but also to identify processes that are not well understood and to propose new model-driven experiments to better understand the system.

## Materials and Methods

### Ethics Statement

We certify that mice were treated in accordance with the guidelines of the University of Navarra (UNAV, Pamplona, Spain).

### Cell Lines and Animals

#### Cell lines

293T cell line derived from human kidney and L929 mouse fibroblast cell line were obtained from American Type Culture Collection, LGC Promochem (Molsheim, France). Both cell lines were cultured in DMEM (Gibco) supplemented with 10% FCS, 100 U/mL streptomycin, 100 mg/mL penicillin and 1% L-glutamine at 37°C and 5% of CO_2_.

#### Animal handling

Animal experiments were performed on 8-week-old female immunocompetent BALB/c mice (Harlan, Barcelona, Spain). Plasmid administration was performed by hydrodynamic injection as previously described [33]. Briefly, plasmids (20 µg of plasmid per mouse) were resuspended in 1.8 mL of saline solution and administered through the tail vein over a short period of time to achieve efficient gene transfer and expression in the liver. Blood samples were collected from retro-orbital plexus, after isofluorane (Abbott, Chicago, IL, USA) anaesthesia.

### Cloning of GFPApo, IFNGFP and IFNGFPApo Plasmids

eGFP plasmid (peGFP) was kindly donated by Dr. Hernandez-Alcoceba. IFNα plasmid (pIFN), ApoAI plasmid (pApoAI) and IFNα bound to ApoAI plasmid (pIFNApoAI) had been previously synthesized in the laboratory [13].

Three different sequences of eGFP were designed for each of the vectors proposed: GFP fused to ApoAI (pGFPApoAI), GFP fused to IFNα (pIFNGFP) and GFP in between IFNα and ApoAI (pIFNGFPApo). PCR amplifications were carried out with the selected primers (listed Table S1). The resulting products were purified (Qiagen, Madrid, Spain), cloned into pcDNA™3.1/V5-His TOPO® TA expression vectors, according to the instructions provided by the manufacturer, and transformed into Top10 bacteria (Invitrogen, CA, USA) for amplification. To proceed with the fusion of eGFP sequences to their vectors (pApo, pIFN and pIA respectively), both plasmids were digested with the appropriate enzymes (New England Biolabs, UK) for each case. Afterwards, the open plasmids and their purified inserts were fused using T4 DNA ligase High Concentration and 2X Rapid Ligation Buffer (Promega, Madison, WI, USA). New plasmids were kept at −20°C until their use.

#### pGFPApo

sense primer FwspGFP, introducing a signal peptide, and antisense primer RvAscIGFP, introducing the restriction site for the AscI enzyme (GGCGCGCCC) in 3′ end of eGFP were designed and used as templates for eGFP amplification. peGFP and pApoAI were digested independently with AscI and BstBI enzymes and cloned as previously described.

#### pIFNGFP

sense primer FwAscIGFP, introducing the restriction site for the AscI enzyme (GGCGCGCCC) in 5′ end of eGFP, and antisense primer RvTaaGFP, were designed and used as templates for eGFP amplification. peGFP and pIFN were digested independently with AscI and BstBI enzymes and cloned as previously described.

#### pIFNGFPApo

sense primer FwAscIGFP and the antisense primer RvAscIGFP previously discussed, were designed and used as templates for eGFP amplification. peGFP and pIFNApo were digested independently with AscI enzyme and cloned as previously described.

### Evaluation of pGFPApo, pIFNGFP and pIFNGFPApo

#### Fluorescence and confocal microscopy

GFP fluorescence was evaluated through fluorescence microscopy (Leica Microsystems, Germany). Briefly, 2×10^5^ 293T cells per well were seeded in 6-well-plates (Cellstar®, Greiner bio-one, Germany). When 80% confluence was achieved, cells were transfected with one of the three plasmids in presence of PEI 25000 kDa (PolyScience, IL, USA). Fluorescence was directly analysed 24 h after transfection.

To study protein location inside the cell, 24 hours after transfection with pGFPApo, pIFNGFP or pIFNGFPApo cells were rinsed and incubated in presence of BODIPY TR 5 µM (Invitrogen, Carlsbad, CA, USA) 30 min at 4 °C. Subsequently, cells were rinsed with cold cell medium and incubated for another 30 min before confocal microscopy analysis. Volocity software was used to evaluate GFP and BODIPY colocalization.

### Determination of IFNα Activity

2×10^5^ 293T cells per well were seeded in 6-well-plates (Cellstar®, Greiner bio-one, Germany) and cells were transfected as previously described. After 24 h, supernatant containing the proteins expressed by the cells was collected to determine the capacity of serial dilutions of supernatants to protect L929 fibroblast cells against the encephalomyocarditis virus following the protocol detailed in [13].

### Detection of IFNα and ApoAI in the Lipoprotein Fractions of Plasma

24 hours after hydrodynamic administration of the pApoAI, pIFNGFP and pIFNGFPApo plasmids, blood samples were taken and very low density lipoprotein (VLDL), low density lipoprotein (LDL), high density lipoprotein (HDL) and lipoprotein depleted plasma (LDP) fractions were isolated from pool plasma samples by floating after sequential differential ultracentrifugations in sodium bromide gradient [40].

Levels of IFNα in each of the fractions were measured by ELISA in NUNC™ maxisorp 96-wells plates (Bioreba, Germany). RAMMA-1 (PBL) was used as anti-mIFNα antibody and HRP-conjugated donkey α-rabbit IgG (Southern Biotechnology, Birmingham, AL, USA) as secondary antibody. Absorbance was read at 450 nm and corrected 540 nm.

Detection of ApoAI was performed by Western Blotting analysis. Lipoprotein fractions were separated by SDS-PAGE (10%) and transferred to a nitrocellulose membrane (Whatman, Maidstone, UK). Goat monoclonal antibody against mApoAI (Santa Cruz Biotechnology,CA, USA) and HRP- conjugated anti-goat IgG were used to detect ApoAI presence and ECL Plus Western Blotting Detection Reagent (Amersham, Barcelona, Spain) was used to reveal the membrane.

### Sampling Collection and Determination

To study the evolution over time of IFNGFP and IFNGFPApo in three main compartments: blood, brain and liver, plasmids were administered to mice as previously described. At 6, 9, 13, 24 and 48 hour time points, blood samples were collected and animals were sacrificed (n = 6–8 mice per point and group obtained from two independent studies). In a third independent study, blood samples at earlier times (1, 3 and 7 hours) were obtained (n = 3 mice per group).

Blood samples were centrifuged 5 min at 9100xg twice and serums were kept at −20°C until their use. Half of each organ was homogenized in distilled water supplemented with a cocktail of protease inhibitors (Complete® EDTA-free, Roche, Barcelona, Spain) and centrifuged to separate proteins from tissue. Supernatants were kept at −80°C until their use. The other half was conserved for RNA extraction.

GFP protein in serum, liver and brain was quantified by GFP ELISA Kit (Cell Biolabs, Inc. Ca, USA) following manufacturer’s instructions.

GFP levels in liver and brain were normalized using the total protein content in the organ, calculated by Bradford analysis. Normalization between experiments was also performed to account for the inter-experiment variability.

2′-5′ OAS, and ISG15 interferon stimulated genes (ISGs) were determined as a measure of IFN activity. mIFNα mRNA was also measured as an indicator of plasmid expression. In order to perform the dynamic analysis, total RNA from mice livers and brains was isolated using TRI reagent (Sigma, Dorset, UK). The concentration and purity of samples were determined by absorbance at 260 and 280 nm, with background correction at 320 nm in a spectrophotometer. RNA was treated with DNase I and retrotranscribed to cDNA with MMLV RT in the presence of RNase OUT (all the reagents from Invitrogen, Carlsbad, CA, USA). The reaction was incubated for 1 hour at 37°C, denatured for 1 minute at 95°C and taken to 4°C. The samples were used immediately for qPCR or stored at −20°C.

Primers for quantitative real time RT-PCR are listed in Table S1. Murine actin was used to standardize gene expression, given that its mRNA levels remained constant with all treatments at several time points (data not shown). The mRNA values were represented by the formula 2ΔCt, where ΔCt indicates the difference in the threshold cycle between mActin and the target genes (all reagents from BioRad, Hercules, CA, USA).

### Data Analysis

The following main processes after administration of plasmids by hydrodynamic injection were described based on a semi-mechanistic computational model using ordinary differential equations (see sets of equations 1–12): (i) hepatic transcription from pDNA to mRNA, (ii) translation of mRNA to proteins in the liver and kinetics of proteins to serum, (iii) protein brain distribution, and (iv) dynamics of protein induced gene expression in liver and brain.

Expression levels of mRNA, GFP levels of protein measured in liver, serum and brain, and interferon stimulated genes in brain and in liver of both molecules (IFNGFP and IFNGFPApo) were used to develop the model. The model was developed sequentially in different steps (see below), and at each step all data available were fitted simultaneously. During the analysis, data were logarithmically transformed. For each measured response, the discrepancy (ε_j_) between an observation obtained at time j^th^ (Y_obs,j_) and the corresponding prediction from the model (Y_PRED,j_) was accounted for as follows: Y_obs,j_  =  Y_pred,j_ x (1+ε_j_). The set of εs forms a random variable with mean 0 and variance equal to ω^2^, which is also estimated as another (random) parameter in the model.

The software NONMEM VI [41] was used during the mathematical analyses. Selection between different model candidates and performance of the selected models was made on the basis of different criteria: (i) precision of parameter estimates, (ii) the minimum value of the objective function value provided by NONMEM (MVOF) and approximately equal to −2xlog (likelihood) (-2LL) and (iii) agreement between observations and model predictions. Differences between two hierarchical (nested) models were compared with a χ^2^ distribution in which a decrease in the MVOF (dMVOF) of 3.84, 6.63 or 10.83 points was considered significant at the 5%, 1% or 0.1% level respectively, for one extra model parameter [41]. Non nested models were compared using the Akaike information criteria (AIC) calculated as Nxlog(RSS/n)+2xNp, where N is the number of data points, RSS the residual sum of squares and Np is the number of parameters in the model [42]. The models with the lowest value of AIC, given that precision of model parameters and data description was adequate, were finally selected.

Exploratory analysis of the data and model evaluation graphs was performed with Prism 5 (GraphPad Software, Inc) and R (R-project).

### Model Building

Figure 4 represents schematically the integrated model finally selected to describe all data available in the current study. In the following, the main modelling assumptions and a detailed mathematical representation of the final model is provided.

#### (i) Hepatic transcription from pDNA to mRNA

After plasmid administration by hydrodynamic injection, almost all the plasmid is located into the liver, where it is expressed [33]. For modelling purposes, it was assumed that drug (plasmid) was directly administered into the liver.

Kinetics of mRNA was modelled simultaneously for both molecules, fitting a model previously developed by Berraondo *et al* [28] where different degrees of active DNA (DNA and DNA’), both able to induced mRNA synthesis, although with different transcription rate constants (k_S1_ and k_S2_ respectively), coexisted in the nucleus after pDNA internalisation. Equations 1–3 represent the model for the dynamics of hepatic transcription from pDNA to mRNA.


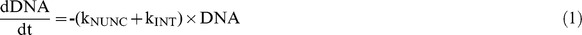



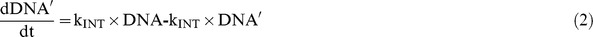



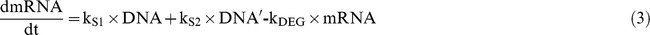


Since information about the effective amount of pDNA reaching the liver was not available, an arbitrary value of 1 was assumed. The amounts of DNA, DNA’, and mRNA at the moment of the pDNA injection (initial conditions) were 1, 0, and 0, respectively. k_NUNC_ and k_DEG_ are the first order elimination rate constants of DNA and mRNA respectively, k_INT_ is the first order rate constant of conversion and degradation of DNA’, and k_S1_ and k_S2_, first order rate constants of expression of mRNA from DNA and DNA’, respectively.

#### (ii) Translation of mRNA to proteins in the liver and kinetics of proteins to serum

To account for the levels of proteins in the liver, as a result of hepatic protein expression, and their release to systemic circulation, parameters k_NUNC_, k_DEG_, k_INT_, k_S1_ and k_S2_ were fixed to those obtained in the previous step. Liver and brain protein density was considered to be 1 mg/mL, and volume of distribution was assumed to be 1 mL for both molecules.

A simultaneous fit was initially attempted for both proteins. However, different dynamics were observed, and therefore, independent models were developed for IFNGFP and IFNGFPApo. The set of ordinary differential equations represented by expressions 4 to 7 were used to describe the time courses of IFNGFP levels in liver and serum (lIFNGFP, and sIFNGFP, respectively), where k_S_ and k_D_ are the hepatic first order rate constants of synthesis and degradation of the protein, respectively and k_TRAN_ represents the first order constant rate of transit accounting for the delay in the synthesis of liver protein from mRNA;. The parameters k_ls_, k_sl_ and k_sb_ represent the rate constants of distribution between the liver (l) and serum (s), and serum and brain (br) compartments.


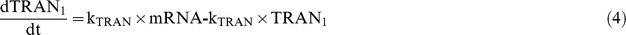



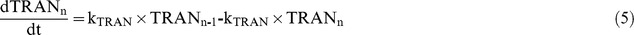



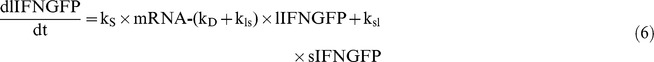



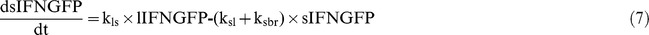


In the case of IFNGFPApo protein, apart from the transit compartments described by eq 4 and 5, a peripheral distribution compartment (p) was required to describe the kinetics of the protein in serum.


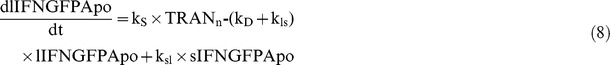



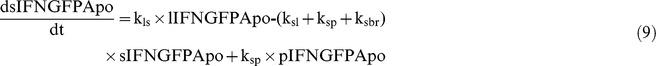



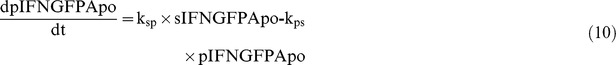


where k_ls_, k_sl_, k_sb_, k_S_ and k_D_ represent the same processes as for IFNGFP case, but with different values. k_sp_ and k_ps_ correspond to the distribution rate constants between central serum compartment and peripheral compartment.

#### (iii) Protein brain distribution

Brain levels of proteins (brIFN standing for brIFNGFP or brIFNGFPApo) were described using a model in which input from the serum and disappearance from the brain compartments were described through a first order rate process represented by the rate constants k_sbr_ and k_E_, respectively. In equation 11, the model corresponding to brIFNGFP and brIFNGFPApo is shown.


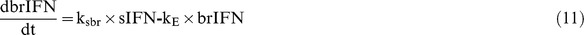


During the analysis of the brain protein levels, the predicted time profiles of both proteins in serum obtained from the previous sub-models (equations 1–10) were used.

#### (iv) Dynamics of protein induced gene expression in liver and brain

The profiles of the brain and liver levels (brISGs and hISGs, respectively) of the following ISGs, OAS and ISG15, were described using a model that assumes that (i) the ISGs synthesis is mediated by the (model predicted) levels of the corresponding proteins in liver or brain, and (ii) ISGs degradation occurs through a first order process (see equation 12), where parameters k_S_ISG_ and k_D_ISG_ represents the first order process of synthesis and degradation, respectively.


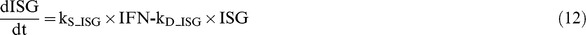


Kinetic and dynamic processes (i.e., synthesis, degradation, distribution) are represented in equations 1–12 as linear processes; however, during model building non-linear models were also explored and tested for model improvements.

## Supporting Information

Figure S1 **Comparison of model performances.** Model predictions against IFNGFPApo observation (points) when no transit compartments (dashed line), or transit compartments with (solid lines) or without peripheral serum distribution (dotted line) are evaluated in liver (**A**) or serum (**B**).
(TIF)

Figure S2 **Mean liver model predictions for IFNGFP (solid line) and IFNGFPApo (dashed line) compounds. Protein levels are expressed in pg/mL.**
(TIF)

Figure S3 **Dynamic of mRNA (gene expression units), liver transit compartments (arbitrary units) and hepatic IFNGFP (left panel) or IFNGFPApo levels (right panel) (pg/mg protein).**
(TIF)

Table S1 List of primers used for PCR and qRT-PCR.
(DOC)
