# Supplementary figures and images for: Kinetic and Dynamic Computational Model-Based Characterization of New Proteins in Mice: Application to Interferon Alpha Linked to Apolipoprotein A-I
Source: PLoS One. 2012 Jul 27;7(7):e42100. doi: 10.1371/journal.pone.0042100 (PMC3407104; doi:10.1371/journal.pone.0042100)

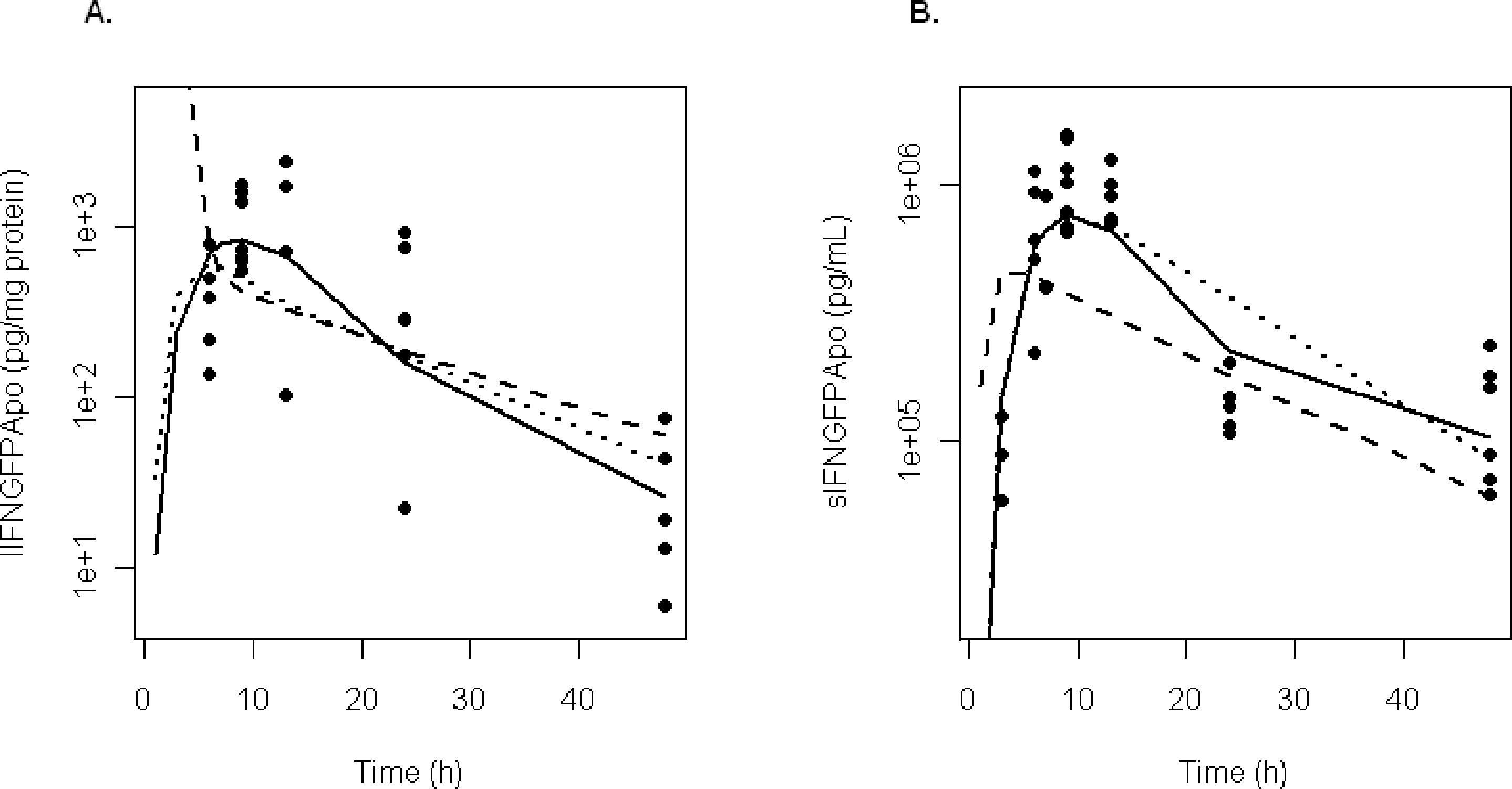

Supplement: Figure S1 — Comparison of model performances. Model predictions against IFNGFPApo observation (points) when no transit compartments (dashed line), or transit compartments with (solid lines) or without peripheral serum distribution (dotted line) are evaluated in liver (A) or serum (B). (TIF) [file pone.0042100.s001.tif]

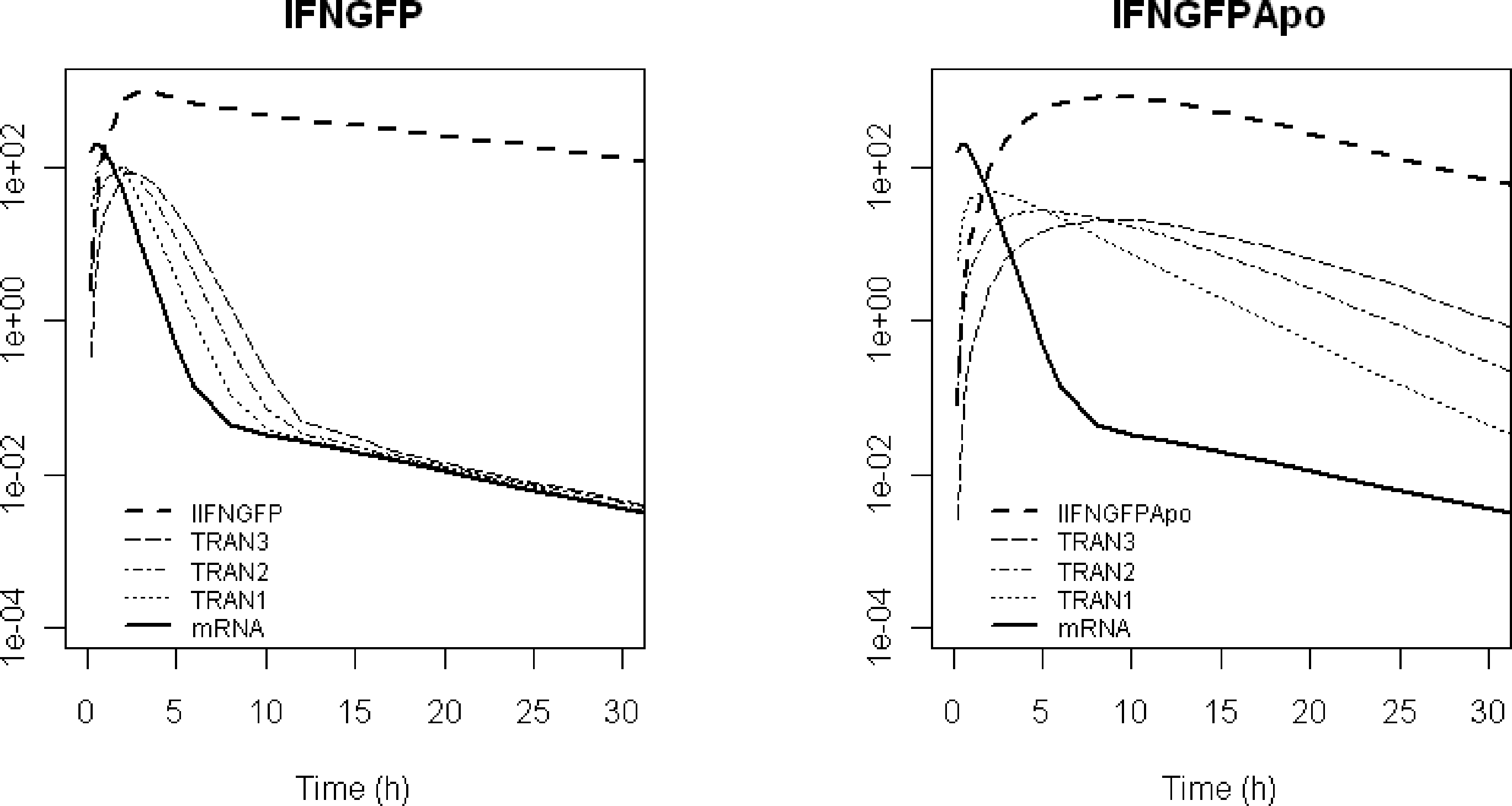

Supplement: Figure S3 — Dynamic of mRNA (gene expression units), liver transit compartments (arbitrary units) and hepatic IFNGFP (left panel) or IFNGFPApo levels (right panel) (pg/mg protein). (TIF) [file pone.0042100.s003.tif]
