# Supplementary material for: Kinetic and Dynamic Computational Model-Based Characterization of New Proteins in Mice: Application to Interferon Alpha Linked to Apolipoprotein A-I
Source: PLoS One. 2012 Jul 27;7(7):e42100. doi: 10.1371/journal.pone.0042100 (PMC3407104; doi:10.1371/journal.pone.0042100)
Supplement: Table S1 — List of primers used for PCR and qRT-PCR. (DOC) [file pone.0042100.s004.doc]

**Table S1:** List of primers used for PCR and qRT-PCR

| **Primer name** | **Sequence** |
| --- | --- |
| FwspGFP | 5’-ATGGCTAGGCTCTGTGCTTTCCTGATGGTCCTGGCGGTGCTGAGCT  ACTGGCCAACCTGCTCTCTAGGAGTGAGCAAGGGCGAGGAG C- 3’ |
| RvAscIGFP | 5’-GGCGCGCCCTTGTACAGCTCGTCCATG -3’ |
| FwAscIGFP | 5’- GGCGCGCCTGTGAGCAAGGGCGAGGAGC - 3’ |
| RvTaaGFP | 5’- TTACTTGTACAGCTCGTCCATG -3’ |
| qPCRFwIFN | 5'-TCTYTCYTGYCTGAAGGAC-3' |
| qPCRRvIFN | 5'-CACAGRGGCTGTGTTTCTTC-3' |
| FwISG15 | 5'GATTGCCCAGAAGATTGGTG-3’ |
| RvISG15 | 5'TCTGCGTCAGAAAGACCTCA-3’ |
| Fw25OAS | 5'-ACTGTCTGAAGCAGATTGCG-3' |
| Rv25OAS | 5'-TGGAACTGTTGGAAGCAGTC-3' |
| FwmActin | 5'CGCGTCCACCCGCGAG-3’ |
| RvmActin | 5'CCTGGTGCCTAGGGCG-3’ |
